# A Thermo-Mechanical Stress Based Fatigue Life Evaluation of a Mine Hoist Drum Brake System Using COMSOL Multiphysics

**DOI:** 10.3390/ma15196558

**Published:** 2022-09-21

**Authors:** Sorin Mihai Radu, Florin Dumitru Popescu, Andrei Andraș, Zoltán Virág, Ildiko Brînaș, Manuel-Ionuț Draica

**Affiliations:** 1 Department of Mechanical, Industrial and Transport Engineering, University of Petroșani, 332009 Petroșani, Romania; 2 Institute of Mining and Geotechnical Engineering, University of Miskolc, H-3515 Miskolc, Hungary

**Keywords:** Matake, Findley, fatigue life evaluation, mine hoist, drum brake system, COMSOL Multiphysics, thermomechanical stress

## Abstract

In this study, the fatigue usage factors for Findley and Matake stress-based criteria were determined in the case of an MK5×2 mine hoist drum brake system subjected to cyclic maneuver braking. The study was conducted for this type of brake system, because the majority of mine hoists in Romanian mines are equipped with this brake type, being in operation for several decades. A geometric model of the brake was built using SolidWorks and imported in COMSOL Multiphysics to perform thermo-mechanical simulations. Based on the deformations and von Mises stresses determined by the thermomechanical simulation and, considering the calculated endurance limits of the brake system materials, Matake and Findley fatigue life evaluation simulations from COMSOL’s fatigue module were conducted. The results show that the highest fatigue is expected on the drum lining surface towards the exit point from under the brake shoe in both cases, and the values of the usage factor of 0.307 (Findley) and 0.401 (Matake) are both under the critical value 1, meaning that the stress limit has not been exceeded for the brake system components and, thus, failure is not expected. Simulations were conducted considering an estimated 1.06 × 10^5^ cycles during one year, more than both the usual service/replacement interval of the friction components of the brake, and the period of mandatory technical inspections imposed by regulations.

## 1. Introduction

**Figure 1:**
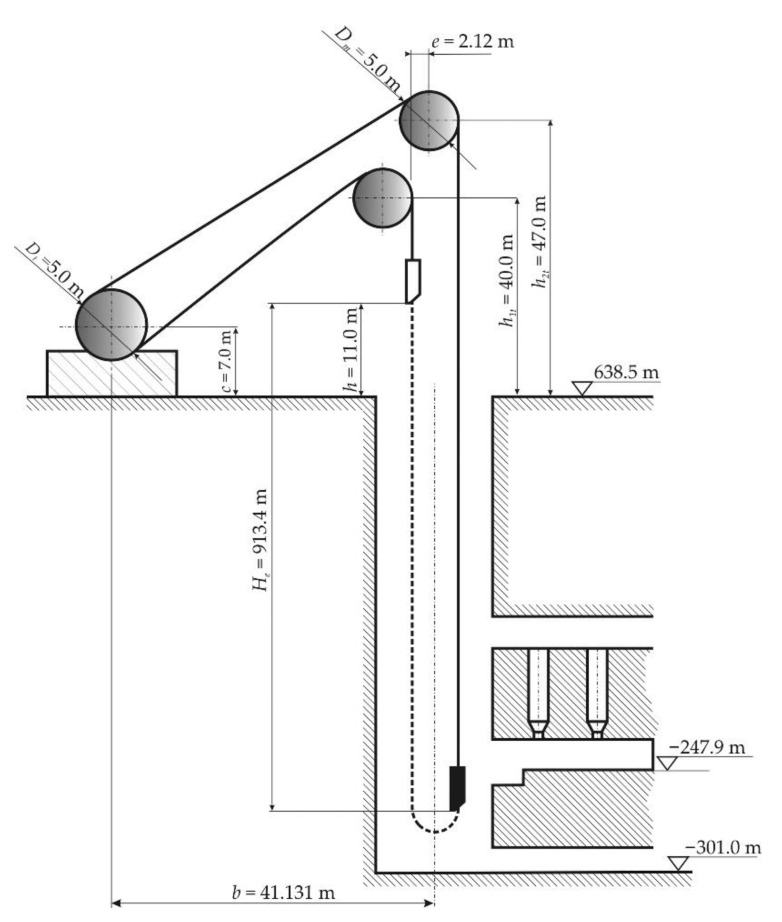
General layout of a friction mine hoist [3].

One of the most important activities in the underground mining operations is the transport of people, material, and ore between the surface and the underground. In order to perform this task, mine hoist systems are used. These systems consist of a series of components, namely the vertical shaft, headframe, ropes, conveyances, headgears and sheaves, and the hoists. There are several standard configurations, including drum hoisting systems, Blair multirope hoisting systems, and Koepe (or friction) hoisting systems [1]. The latter is based on the bollard friction principle, and the rope is not attached to the drum. The conveyances are moved reciprocally up and down the shaft, and in order to ensure the transmission of friction forces, the system usually uses tail ropes. These are fixed underneath the conveyances to balance out the loads in the shaft hoist ways and form a closed system [2]. Koepe or friction hoist systems are the most used in Europe and also in Romania, and are characterized by the maximal depth of shaft, the depth of the loading/unloading points, the diameter of the head sheaves, and the placement of the drive wheel, as shown in Figure 1 [3]. 

**Figure 2:**
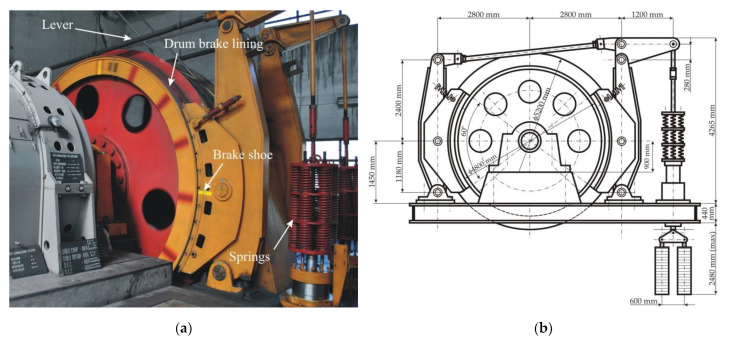
The MK5×2 hoisting machine: (**a**) Photo of the drum brake system; (**b**) detailed drawing of the drum brake system [4].

The brake systems used in Romanian MK5×2 model friction hoisting machines are of the drum-and-shoe type [4], as shown in Figure 2a. This type of brake consists of a drum lining placed on each side of the rope wheel circumference, and two pairs of brake shoes lined with friction material, situated on the drum lining diametrically opposite, together with a series of springs and levers that are actuating the brake, as shown schematically in Figure 2b. The features of the MK5×2 mine hoist drum brake system are described in the technical documentation from the manufacturer [5]. These features are presented in Table 1 and were used to create the 1:1 scale geometric model, built as described in Section 4.

During the braking process, the levers are squeezing the brake shoes against the lining of the rotating drum. Because of friction, the rotation speed is decreased up to a complete stop when required. The friction produces a lot of heat, and this phenomenon is cyclic during the life of the mine hoist.

Several researchers investigated the fatigue of various brake systems. In the case of rail vehicles brakes, Dufrénoy et al. [6] analyzed the fracture mechanisms in TGV disc brakes using a combination of experimental crack analysis and numerical simulations. The fatigue life assessment of the brake disc of a railway vehicle was performed by Kim et al. [7] in his study, by applying the linear relation between the temperature and the circumferential stress. Wu et al. [8] used XFEM methods to evaluate the fatigue life and safety domain for a high-speed railway brake disc. A complex review of methods of railway stresses, temperature, and fatigue simulations was conducted by [9], based on 49 publications in the last decade. Additionally, for railway brakes, Yevtushenko et al. [10] developed a new FEM investigation method with simplified spatial models of friction heating.

Regarding the fatigue studies conducted for automotive brakes, Zhang’s paper [11] uses a combination of ABAQUS, Fe-safe, and nCode software to obtain the location, life, and crack length of fatigue crack initiation on the brake subjected to thermomechanical stress and, thus, obtains a fatigue life prediction method of the brake disc. Choi and Lee [12] found that the circumferential stress produced during rotation led to the failure of the solid automobile brake disc. Afzal et al. compiled an extensive review of the numerical and experimental fatigue studies in the case of solid and ventilated disc brakes with automotive applications [13]. Ramesh et al. [14] used ANSYS Software to investigate the influence of coatings on the mechanical properties and life of brake components.

Larger brakes were also researched in terms of fatigue. Gigan et al. studied the cyclic response and the fatigue life of heavy vehicle brake discs made from grey cast iron, using four different models of fatigue life assessment [15]. The fatigue of a heavy truck brake disc material was analyzed by Akop et al. who concluded that the thermally induced stresses developed in the disc caused cracking and fatigue stress in radial and axial directions [16]. Fatigue life was calculated by Rouhi et al. based on a 3D coupled thermomechanical FE analysis for both the braking and cooling phases of a heavy vehicle brake [17]. In the study [18] of Korba et al., for two thrust plates of the multiple-disc aircraft brake unit, the lifetime analysis was performed using numerical methods (i.e., Nastran finite elements software). Ramadan et al. performed [19] simulations of thermally induced fatigue in a multi-disc brake during aircraft braking. Furthermore, COMSOL Multiphysics was used by Kamal et al. [20] to evaluate the fatigue crack retardation of composite materials used in industrial brakes, by Elsheikh et al. [21] to demonstrate a new temperature field reconstruction method proposed for thin-wall disc, and also by Jeong et al. [22] for a series of simulations regarding ECBs suitable for small wind turbines.

Apart from fatigue studies conducted on various brakes used in commercial or industrial vehicles, to the best of our knowledge no such approach regarding fatigue was performed in the case of very large industrial applications, such as mine hoist brakes, so this is the novelty that this study brings.

In the case of mine hoists, research of fatigue evaluation has concentrated on the fretting fatigue of the hoisting rope [23], steel wire fatigue due to bending over the sheaves [24], and the tribo-fatigue because of the rope and friction pulley contact [25,26]. Additionally, the cage steel guide fatigue [27], the fatigue endurance of the load bearing elements [28], the fatigue damage accumulation of the winding drum [29,30], and the headframe structure improvement to sustain fatigue [31,32] were studied but, as far as we are aware, no previous research regarding the fatigue life evaluation of the components of mine hoist brakes was published.

**Figure 3:**
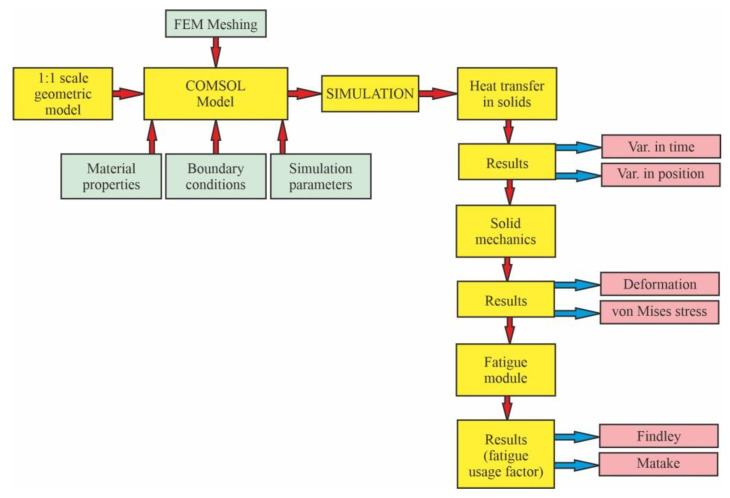
The step by step approach used in the research.

Bearing this in mind, the objective of the present study is to investigate what happens to the components of mine hoist brakes in terms of fatigue after an imposed period of time. Based on the hoisting diagram of one hoisting cycle, the total number of braking events during one year was calculated. A simulation was run in COMSOL Multiphysics 5.3 [33] to determine the thermal behavior of the brake during a hoisting cycle, the shape and direction of the thermally induced deformations, and the stresses generated by these deformations. Finally, considering the results of the thermomechanical simulation and using the *Fatigue Module* [34] of COMSOL Multiphysics, the Findley and Matake fatigue usage factors were determined as being under the critical value of 1. The step by step approach is shown schematically in Figure 3.

## 2. Theoretical Considerations about the Kinematics of the MK5×2 Mine Hoisting Installation

The operation of the hoisting installations has a cyclical character, based on a hoisting diagram. The shape of the hoisting diagram and the ratio between its phases influence the magnitude of the driving power, the electrical and mechanical losses in the hoisting installation subassemblies, the energy consumption, the overall efficiency of the installation, and its productivity. The actual shape of a hoisting diagram is determined by several factors. Firstly, the transport distance determines both the number of phases of the hoisting diagram and the maximum transport speed. The operation mode of the hoisting installation influences the shape of the hoisting diagram [2]. Other factors which are decisive in determining the shape of the hoisting diagram are the technological conditions and also those related to the operational safety, both of which influence the number of phases and the limitations of hoisting velocity and acceleration.

**Figure 4:**
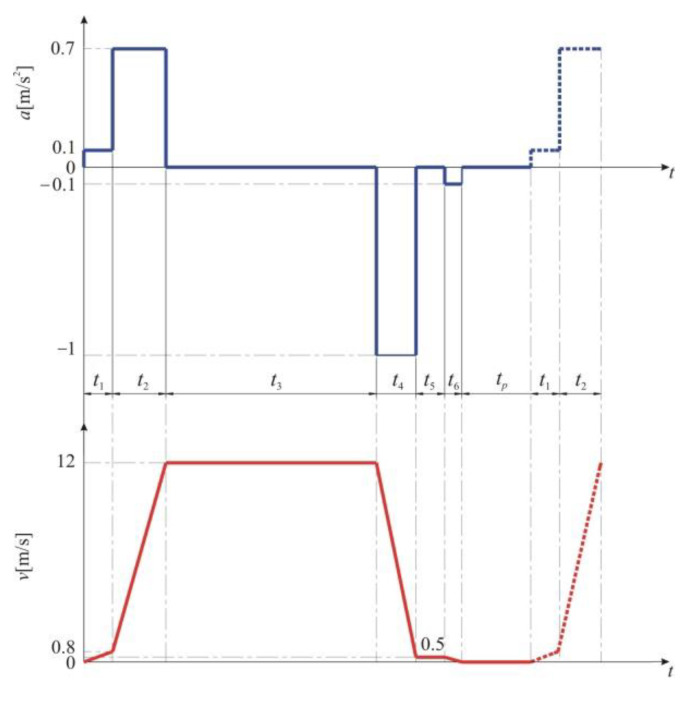
Characteristic six-phase hoisting diagram of the MK5×2 type hoisting installation.

Theoretically, the hoisting diagrams are drawn starting from the variation of the acceleration, which in turn is determined by the mode of operation of the installation. Knowing that the velocity is the acceleration integrated in relation to time, the mode of variation of the transport velocity over the different intervals of a transport cycle is subsequently determined. Finally, knowing that the space traveled is the velocity integrated in relation to time, the distances corresponding to the phases of a transport cycle can be determined. The six-phase hoisting diagram shown in Figure 4 is characteristic of the MK5×2 type hoisting installation with the parameters presented in Table 2 [3].

In order to maintain the dynamic stresses at a low level, and also because of the technological operating conditions due to velocity limitations, the acceleration *a_1_* during the period *t*_1_ must not exceed the maximum value of 0.1 m/s^2^. This phase of the hoisting diagram corresponds to a certain space during which acceleration and velocity are limited. During the time interval *t*_2_ the acceleration is limited to the value of 0.7 m/s^2^, in order to limit the dynamic stresses. At the end of this second acceleration period, the transport velocity reaches a value of 12 m/s. During the time interval *t*_3_ the transport is carried out with constant velocity. The hoisting installation is stopped in two stages. Thus, during the time interval *t*_4_, the deceleration is −1 m/s^2^, when maneuver braking is performed until the velocity reaches 0.5 m/s. Once this value is reached, the brake is no longer applied, which means that the transport is carried out at a constant speed during time interval *t*_5_. The velocity during this interval is imposed by the technological conditions of operation, with the conveyances physically moving in the unloading area. The time interval *t*_6_ corresponds to the final braking of the hoisting installation, with a deceleration of −0.1 m/s^2^ until zero velocity. During the time interval *t*_p_, the unloading of the conveyances at the mine surface takes place, namely the loading of the underground conveyance and, after that, another extraction cycle starts. From the above, it can be concluded that braking of the hoisting installation takes place in two stages, during the time intervals *t*_4_ and *t*_6_, respectively. The operation is cyclical and, for each extraction cycle, stopping involves the actuation of the brake twice before the transport velocity becomes zero. These repetitive braking periods cause a cyclic transformation of the kinetic energy of the moving masses into heat which causes the heating or cooling of the passive and active parts of the braking system.

From the hoisting parameters described in Table 2, several times, spaces, and velocities can be calculated [3,4] as follows.

The time required to pass the unloading curves can be calculated as follows:

(1) $$t_{1}=\sqrt{\frac{2\cdot h_{1}}{a_{1}}}=8.36\;s$$

Exit velocity at the unloading curves can be calculated as follows:

(2) $$v_{1}=a_{1}\cdot t_{1}=0.836\;m/s$$

Time to accelerate to maximum velocity:

(3) $$t_{2}=\frac{v_{\max}-v_{1}}{a_{2}}=\frac{\left(v_{1}+a_{2}\cdot t_{2}\right)-v_{1}}{a_{2}}=15.94\;s$$

The space travelled during period *t*_2_ can be calculated as follows:

(4) $$h_{2}=v_{1}\cdot t_{2}-\frac{a_{2}\cdot t_{2}^{2}}{2}=102.25\;m$$

The time required for deceleration can be calculated as follows:

(5) $$t_{4}=\frac{v_{\max}-v_{4}}{a_{4}}=11.5\;s$$

Space travelled during period *t*_4_ can be calculated as follows:

(6) $$h_{4}=v_{\max}\cdot t_{4}-\frac{a_{4}\cdot t_{4}^{2}}{2}=71.875\;m$$

Time of constant velocity crawl at the unloading chute can be calculated as follows:

(7) $$t_{5}=\frac{h_{5}}{v_{4}}=4\;s$$

Space travelled during constant maximum velocity *v*_max_ can be calculated as follows:

(8) $$h_{3}=H_{e}-\left(h_{1}+h_{2}+h_{4}+h_{5}\right)=733.78\;m$$

Time of constant velocity hoisting can be calculated as follows:

(9) $$t_{3}=\frac{h_{3}}{v_{\max}}=61.14\;s$$

The loading/unloading time is considered as $t_{p}=20\;s$.

Thus, the total time of one transport cycle is as follows:

(10) $$T=\sum_{i=1}^{6}t_{i}+t_{p}=129.3\;s$$

The total time of braking the hoisting installation is as follows:

(11) $$T_{\mathrm{brake}}=t_{4}+t_{5}+t_{6}=23.86\;s$$

Thus, the simulation time will be as follows:

(12) $$T_{\mathrm{sim}}=T_{\mathrm{brake}}+t_{p}=43.86\;s$$

Theoretically, the maximum number of transport cycles that can be performed during one hour is as follows:

(13) $$N_{c}=\frac{3600}{T}\approx 27\;\mathrm{cycles}/\mathrm{hour}$$

Based on the assumption that the hoisting installation works 24 h a day for 22 days per month, the total number of transport cycles (and brake cycles) during one year can be estimated as follows:

(14) $$N_{y}=N_{c}\cdot 24\cdot 22\cdot 12\cdot C_{\mathrm{ui}}\approx 1.06\times 10^{5}\;\mathrm{cycles}/\mathrm{year}$$

where *C*_ui_ is the coefficient of intensive use, with a value of 0.62 for this type of mine hoist.

## 3. Theoretical Aspects of Low Cycle Fatigue (LCF) and High Cycle Fatigue (HCF)

The fatigue phenomenon has two different regimes, namely low cycle fatigue (LCF) and high cycle fatigue (HCF) [35]. Significant plastic strains on the macroscopic scale that occur throughout each load cycle are a feature of LCF. There is a lowest stress level for some materials, such as many steel and titanium alloys, below which fatigue does not happen no matter how many load cycles are applied. This level is called the endurance limit or the fatigue limit, and these limits are usually of the order of half the ultimate tensile strength. Many materials, such as copper or aluminum, do not appear to have a fatigue limit, but even in the case of materials with no fatigue limit, such values are given. They actually represent the value of the S–N curve at a very large number of cycles, usually 10^8^.

Since the relevant descriptive parameter of LCF is strain rather than stress, low cycle fatigue is usually referred to as “strain-based”. From a physical point of view, strain is also responsible for the damage in HCF but, since HCF occurs in the elastic regime, it is possible to use both strain and stress as the parameter. For practical and historical reasons stress was used, hence, high-cycle fatigue is considered to be “stress-based”.

Usually, a cycle count to fatigue of more than 10^4^ is regarded as a “high cycle”,so the number of cycles/year calculated in Section 2 corresponds [36] to a high cycle fatigue (HCF) regime of the brake.

### 3.1. Fatigue Quantities Definitions

When fatigue fractures occur, the mean stress is also important, as well as the stress amplitude. Compressive stress increases the fatigue life, while tensile mean stress decreases it. The following definitions can be used if σ_max_ is considered the maximum stress and σ_min_ is considered the minimum stress over a cycle:

Stress amplitude: $\sigma_{a}=\frac{\sigma_{\max}-\sigma_{\min}}{2}$; Stress range: $\Delta \sigma =\sigma_{\max}-\sigma_{\min}$; Mean stress: $\sigma_{m}=\frac{\sigma_{\max}+\sigma_{\min}}{2}$; *R*-value: $R=\frac{\sigma_{\min}}{\sigma_{\max}}$.

The R-value is the most common parameter used to describe the level of mean stress. The most common fatigue test is the fully reversed load, when the mean stress is zero and *R* = −1. The second fundamental test is the pulsating test, where the load varies between zero and a maximum value, giving *R* = 0.

### 3.2. Critical Plane Methods

Most of the fatigue criteria in case of multiaxial fatigue [37] are based on the critical plane concept. This is the plane with a certain orientation at the point that maximizes the stress or strain expression in the loaded structure. Various models use different criteria for the critical plane determination, and a successful model must predict both the dominant plane and the fatigue life.

Determining the critical plane requires looking at the load history from every angle conceivable for nonproportional loading, since the critical plane’s orientation is not trivial. This task is generally a computationally heavy one. The default method used in COMSOL’s *Fatigue Module* is to determine the circle that circumscribes all points in the shear stress plane. The choice for the simplified method is made using the *Shear range* search method setting in the *Evaluation Settings* section for the *Strain-Based* and *Stress-Based* nodes [34].

### 3.3. Stress-Based Fatigue Models

Both the Findley and Matake criteria discussed in this subsection and applied in the study are critical plane methods.

The Findley criterion [38] can be defined as follows:

(15) $${\left(\frac{\Delta \tau }{2}+k\cdot \sigma_{n}\right)}_{\max}=f$$

where *f* and *k* are material parameters, σ*_n_* is the largest normal stress on a plane, and Δ*τ* is the maximum shear stress range on the same plane. The critical plane is considered the one that maximizes the left-hand side of the equation.

The ratio between the left-hand side of the Findley criterion and the material parameter *f* is the fatigue usage factor *f*_us_. A fatigue usage factor value below 1 means that the component is loaded below the fatigue limit. For high compressive stress states, the contribution from the normal stress can dominate the criteria, predicting a negative *f*_us_. In those cases, the fatigue usage factor is set to zero.

(16) $$f_{\mathrm{us}}=\frac{{\left(\frac{\Delta \tau }{2}+k\cdot \sigma_{n}\right)}_{\max}}{f}$$

To determine the two parameters *k* and *f* of the material, two fatigue tests with different loading conditions are needed. These can be pure torsion and pure tension, but there are also other options. In the case of axial loading, the following formula is valid:

(17) $$\sqrt{{\left(\frac{\sigma_{\max}-\sigma_{\min}}{2}\right)}^{2}+{\left(k\cdot \sigma_{\max}\right)}^{2}}+k\cdot \sigma_{\max}=2\cdot f$$

In this equation σ_max_ is the maximum and σ_min_ is the minimum stress at the fatigue limit (infinite life). Thus, tests with two different values of *R* can be used to determine *k* and *f*. In the case of a fully reversed torsion test with *τ*_a_ amplitude of the torsional shear stress, the corresponding relation is as follows:

(18) $$\tau_{a}=\frac{f}{\sqrt{1+k^{2}}}$$

If only uniaxial test data with a single *R* value is available, it is possible to estimate *k* from the ratio between the fatigue limits under different conditions for a similar material.

The Matake criterion [39] is quite similar to the Findley criterion, the difference being that the plane with maximum shear stress range is considered as the critical plane, and the maximum normal stress is evaluated on that plane. The expression is as follows:

(19) $${\left(\frac{\Delta \tau }{2}\right)}_{\max}+k\cdot \sigma_{n}=f$$

When the Matake equation becomes negative at high compressive stress states, the fatigue usage factor is set to zero, just as for the Findley criterion.

## 4. Material and Methods

First, the geometric model of the mine hoisting drum brake was created, at true scale, using SOLIDWORKS 2016 x64 Edition. This model was developed in our department by the same authors as a basis for previous work studying thermal behavior during emergency braking [40] and thermal behavior investigations of disc brakes [41]. This geometry was imported in COMSOL Multiphysics 5.3 using the *LiveLink* feature, and the materials and their properties were defined for the drum brake system components, as shown in Table 3. After the choice of materials and the definition of properties, the mesh of the FE is generated. It is a *Physics-controlled* type mesh, with the element size set to *Fine*. The result generated 8506 mesh vertices. The element types and numbers are as follows: tetrahedra (28,489), triangles (17,608), edge elements (2610), and vertex elements (36), with a total of 48 × 10^3^ elements. The total volume of the resulting mesh is 0.6851 m^3^.

Next, the simulation parameters, such as coefficient of friction [42,43], air temperature, wheel radius, and mass of moving parts are defined. Additionally, the initial speed, first and second accelerations, braking times, and step iterations are set, as can be seen in Table 4.

Finally, the surfaces of the drum, the contact surface of the drum and shoe couple, and the surfaces of the brake shoes were defined as *Geometric entity level* of type *Boundary*. The external faces for which the heat exchange takes place by convection have been defined as *Geometric entity level* of the type *Domain*. Additionally, between the drum and shoe and the external surface contact with air, a *Nonlocal Coupling* was defined as being of the type *Integration*, with *Geometric entity level* also being a *Boundary* type.

Based on the phases of the hoisting diagram presented in Section 2 and the initial velocities and times calculated for the studied mine hoist, the velocities are defined (Table 5) for the COMSOL simulation, as functions of braking times previously set in the software.

**Figure 5:**
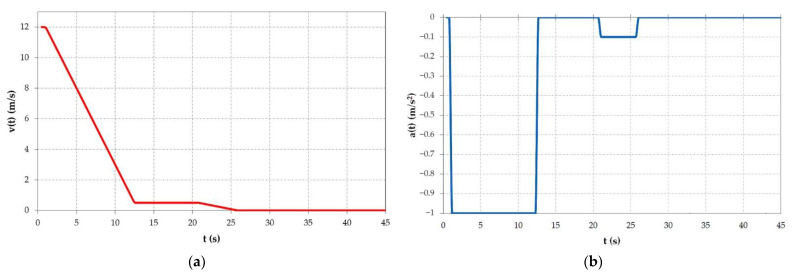
Diagrams obtained in COMSOL for (**a**) velocity and (**b**) acceleration.

Resulting from the functions in Table 5, the diagram of the velocity during the braking phase is obtained as visible in Figure 5a, and the acceleration is automatically determined by derivation of velocity, with a shape as presented in Figure 5b. As expected, both the velocity and acceleration have a similar shape as in the theoretical hoisting diagram.

## 5. Results and Discussion

### 5.1. Results and Discussion for the COMSOL Thermal Simulation Using Heat Transfer Module in Solids

In order to present and discuss the results obtained after the thermal simulation of the maneuver braking of the mine hoist, a series of COMSOL specific data sets are introduced.

**Figure 6:**
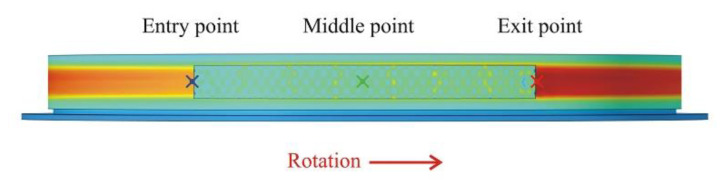
Location of the three points of the Cut Point 3D type.

**Figure 7:**
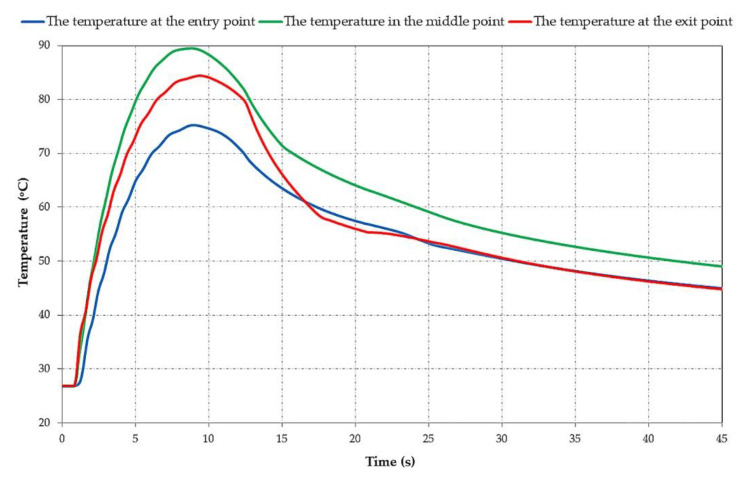
Variation in time of the drum lining surface temperatures for the three points.

The first is a series of three points of type *Cut Point 3D*, as shown in Figure 6, located at the contact between the drum lining and the brake shoe. The first point is located at the entry under the brake shoe; the second point is located at the middle of the brake shoe, while the third point is located at the exit under the brake shoe. These points are chosen to show the variation in time of the surface temperature of the drum lining during braking in the three locations, as plotted in Figure 7.

During the maneuver braking, compared with the emergency braking [40], the highest temperature of approximately 89°C is obtained at the middle point at about 8 s. By analyzing the three graphs it is visible that from the start up to around 8 s, the middle point has a uniform increase while the entry and exit points show a non-uniformity, with the fluctuations visible on the blue and red graphs. This is due to the fact that, for the entry and exit points, there is a slight cooling of the drum lining surface before it enters again under the brake shoe during successive rotations until stopping while, in the case of the middle point, which is permanently under the brake shoe, this convective cooling does not take place. It is visible that in the cooling phase, from around 25 s until the end, the cooling of the exit and entry points has a similar curve, and the temperature of these points is lower than the temperature of the middle point during the whole braking phase. It is important to highlight that the location of the three points was chosen as such, because, in the case of mine hoists, there are significant temperature differences on the drum lining surface between these points. These differences are normal, given the much larger dimensions of the components of the braking system, and the different rotation velocity and braking time in the case of mine hoists as compared to automotive or rail brake systems.

**Figure 8:**
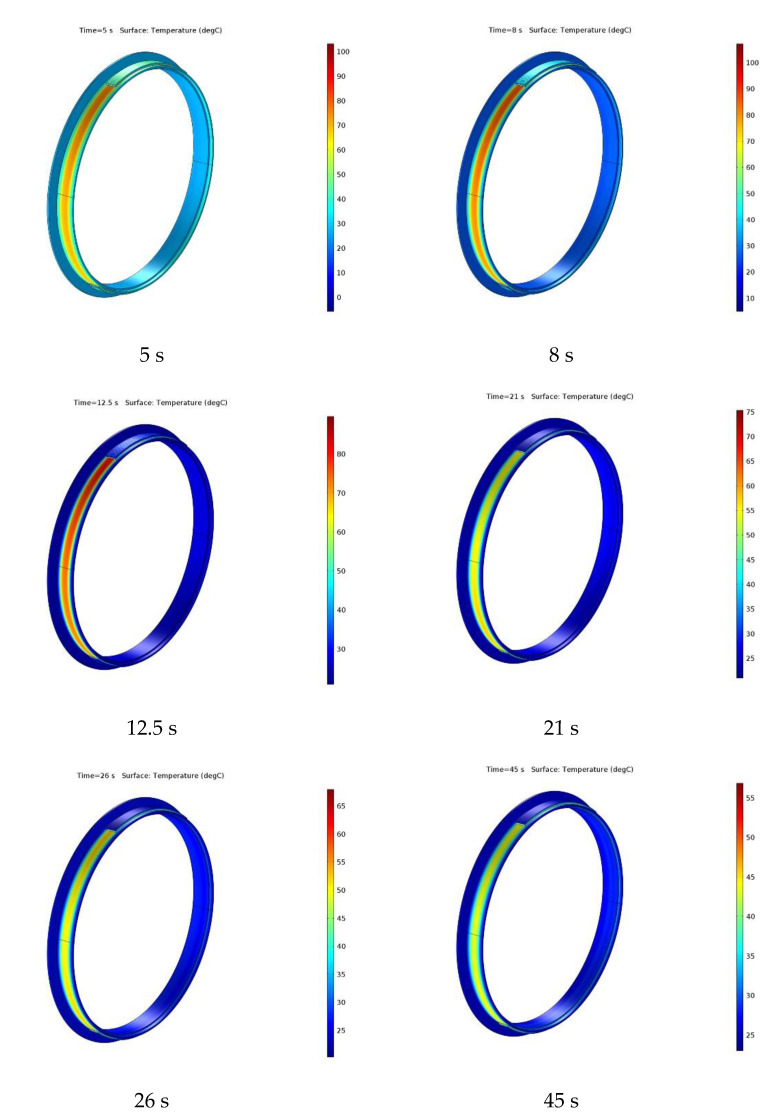
The drum lining surface temperature at different times during braking.

The drum lining surface temperature at different times (5 s, 8 s, 12.5 s, 21 s, 26 s, and 45 s) during the braking is presented in Figure 8 in order to better highlight the aforementioned temperature increase and cooling.

**Figure 9:**
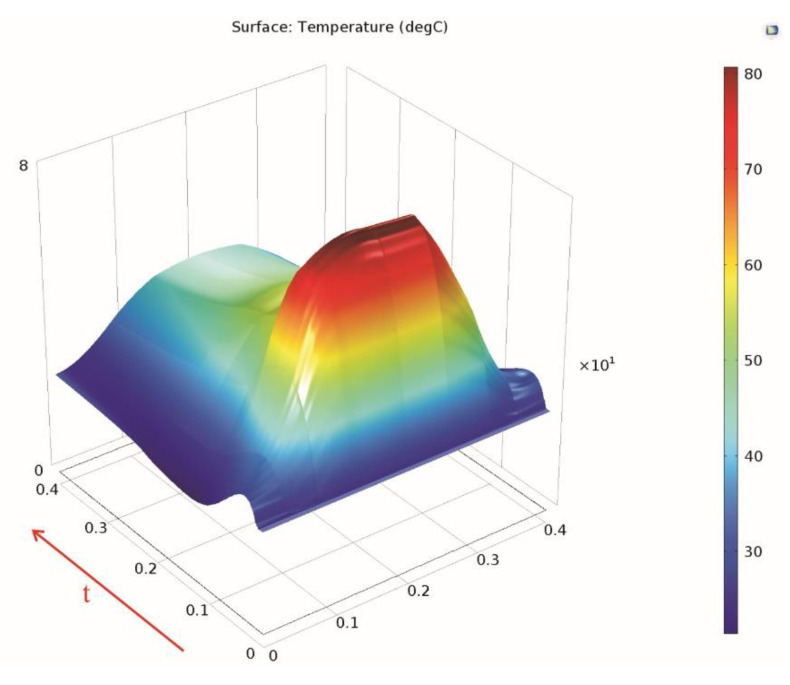
Temperature variation in time along the Cut Line 3D data set.

**Figure 10:**
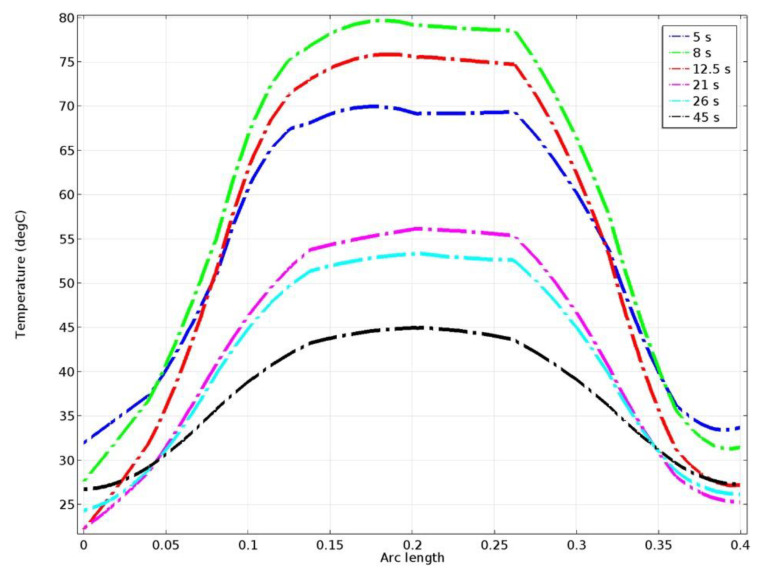
Temperatures along the Cut Line 3D data set, at certain moments of time.

The next specific data set introduced is of type *Cut Line 3D*. This line is drawn transversally through the 3D geometries of the drum and brake shoe. The temperature variation in time along the cut line during the maneuver braking is shown in Figure 9. It is visible that the highest temperatures appear towards the middle of the drum and brake couple section, and a cooling takes place in time. Figure 10 shows the actual temperature along the cut line at specific moments of time (5 s, 8 s, 12.5 s, 21 s, 26 s, and 45 s).

Finally, the last data set is of the *Parameterized Curve 3D* type. This curve is circular, defined by the parametric equation of a circle in relation to its plane coordinates, with a radius equal to that of the drum lining, and a position at the center of the brake shoe and drum line geometry.

**Figure 11:**
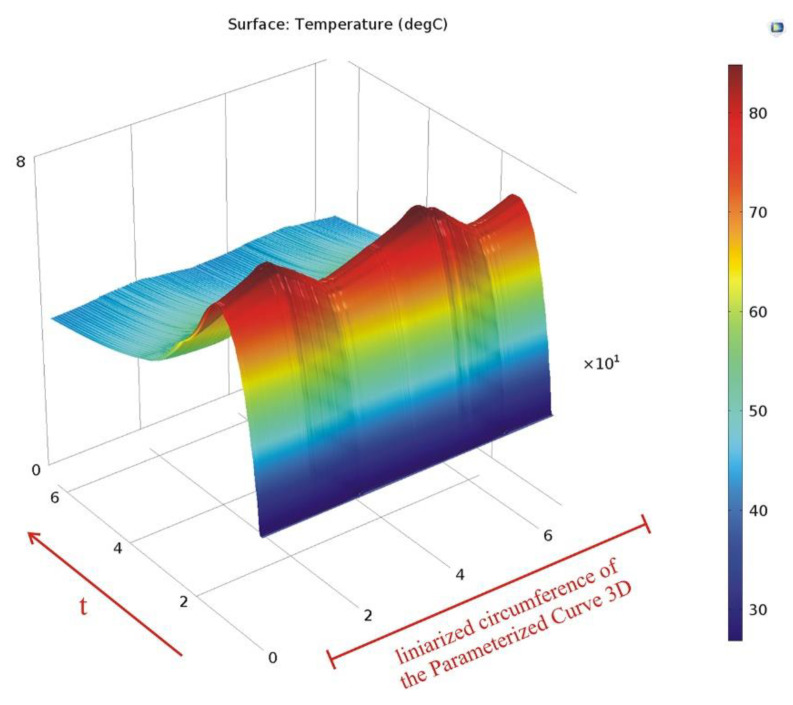
Temperature variation in time along the parameterized curve.

This data set is used to linearly plot the temperature variation in time along this circumference, as shown in Figure 11. The two peaks visible in the graph highlight the drum lining positioned under the brake shoes.

The last two COMSOL data sets introduced in this paragraph will also be used to present the results obtained during the mechanical analysis.

### 5.2. Results and Discussion for the COMSOL Mechanical Simulations Using Solid Mechanics Module

Due to the different thermal expansion coefficients of the materials in the composition of the brake system elements, and the heating–cooling cycles generated during repetitive maneuver braking, thermal stresses occur. Deformations and mechanical stresses also emerge as a result of these thermal stresses. Using the *Solid mechanics* module of COMSOL Multiphysics based on the results of the thermal analyses previously conducted, mechanical simulations were run on the same model in order to determine the deformations and the von Mises stress at time t = 9 s. This moment of time was chosen as it is when the maximum temperature of the middle point is reached.

**Figure 12:**
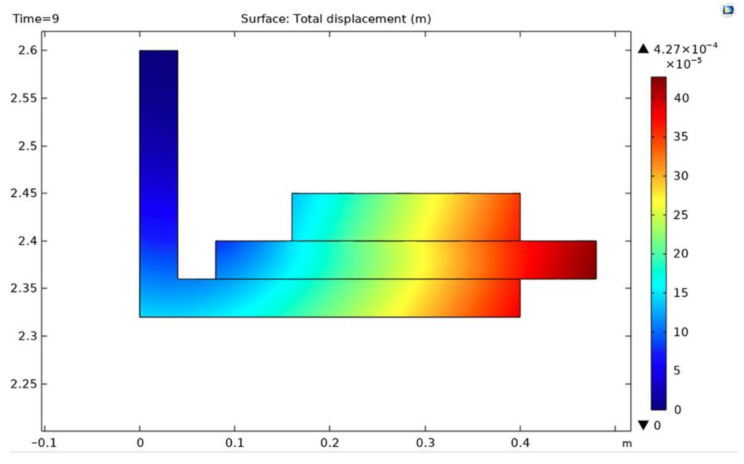
Cross-section of the deformation at t = 9 s.

The maximum deformation of the drum brake lining and shoe couple at this time is 4.27 × 10^−4^ m, situated towards the external edge of the lining. The result is presented in the cross-section in Figure 12.

**Figure 13:**
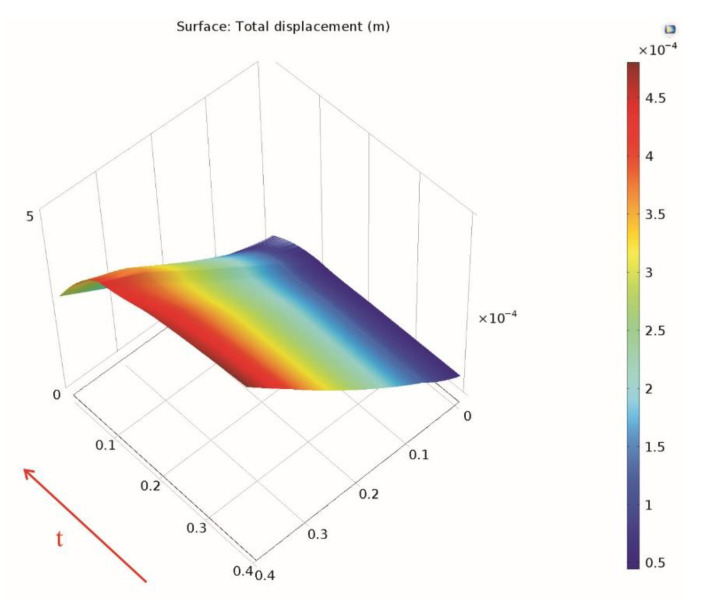
Variation in time of the deformation along the Cut Line 3D data set.

**Figure 14:**
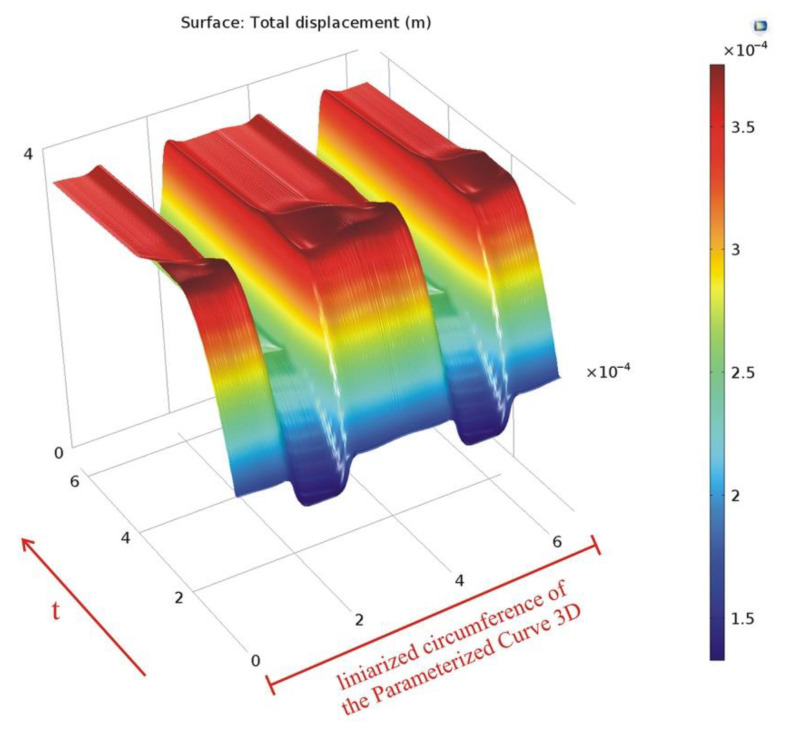
Variation in time of the deformation along the parameterized curve.

The variation in time of the deformation is also presented along the *Cut Line 3D* data set (Figure 13) and along the linearized circumference of the *Parametrized Curve 3D* data set (Figure 14).

**Figure 15:**
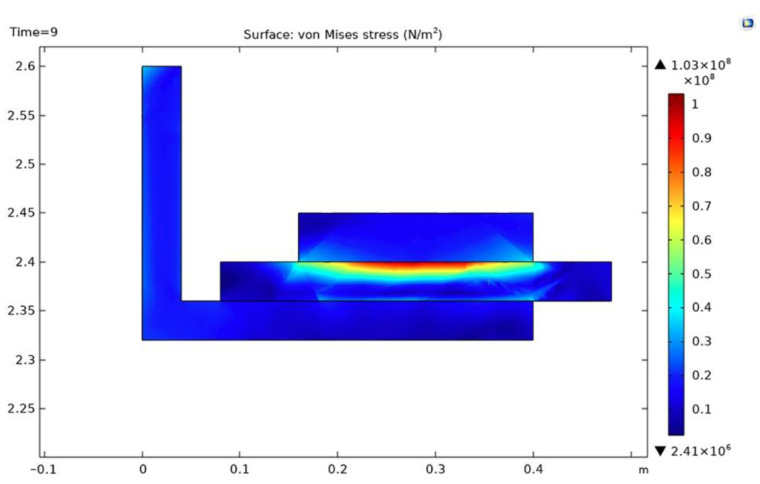
Cross-section of the von Mises effective stress at t = 9 s.

The von Mises effective stress induced in the drum brake lining and shoe couple, at the time t = 9 s, is maximal towards the exterior drum lining surface (Figure 15), where contact with the brake shoe arises; a determined value of 1.03 × 10^8^ N/m^2^ resulted from simulation. This type of stress appears when a certain body subjected to heat and, thus, deformation is not allowed to expand or contract freely.

**Figure 16:**
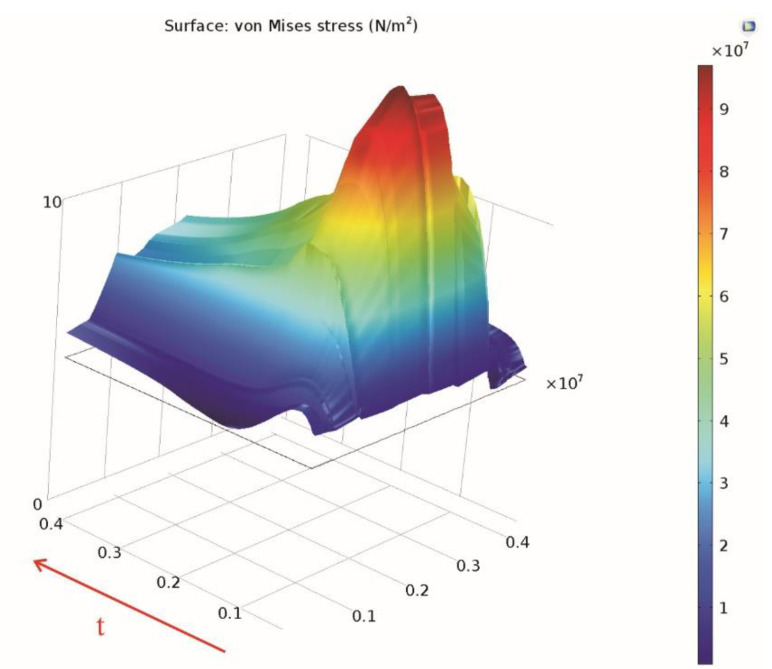
Variation in time of the von Mises stress along the Cut Line 3D data set.

**Figure 17:**
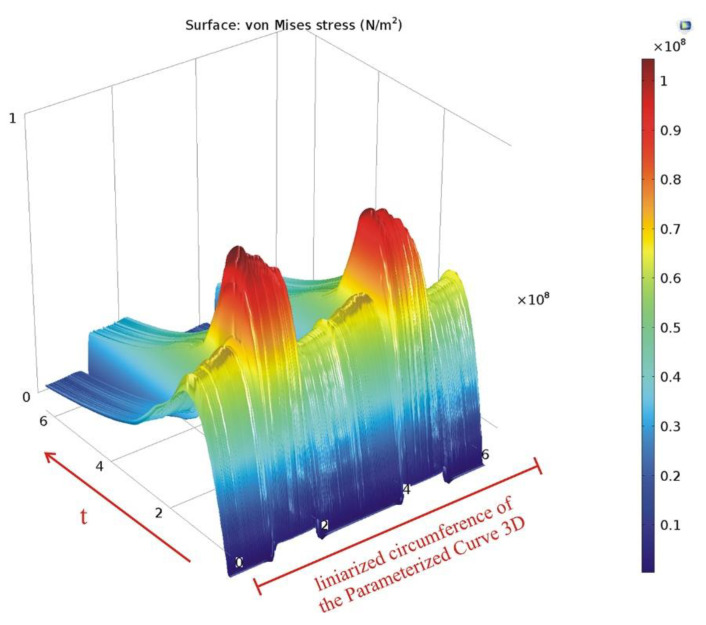
Variation in time of the von Mises stress along the parameterized curve.

The variation of the von Mises stress in time is presented using the data sets introduced, along the *Cut Line 3D* data set (Figure 16) and along the linearized circumference of the *Parametrized Curve 3D* data set (Figure 17).

### 5.3. Results and Discussion for the COMSOL Fatigue Simulations Using Fatigue Module

The fatigue criteria to be applied in the COMSOL fatigue study are Findley and Matake, which are based on the theoretical aspects presented in Section 3, and the actual material properties for the brake system components. The parameters of material [44] are experimentally determined from uniaxial fatigue tests, with test results available [45]. The first one involves reversed tension–compression testing, where the endurance limit is determined by the load amplitude oscillating around zero stress as being σ*_R_*_=__−1_ = 206 MPa. The second test involves testing a material under pure stress with a load that pulses between zero and two times its amplitude and an endurance limit of σ*_R_*_=0_ = 160 MPa. The endurance limit’s denominator displays the test’s *R*-value.

Thus, the Findley parameters in relation to the above endurance limits can be expressed as follows:

(20) $$f_{F}=\frac{\sigma_{R=-1}}{2}\left(k_{F}+\sqrt{1+k_{F}^{2}}\right)$$

(21) $$f_{F}=\frac{\sigma_{R=0}}{2}\left(2\cdot k_{F}+\sqrt{1+4\cdot k_{F}^{2}}\right)$$

By equaling the two equations, the normal stress sensitivity coefficient (*k_F_*) and the Findley limit factor (*f_F_*) are determined in Mathcad, with the values *k_F_* = 0.208 and *f_F_* = 126.57 MPa, respectively. These values are defined as properties of materials (Table 6) for the COMSOL Findley fatigue simulation.

**Figure 18:**
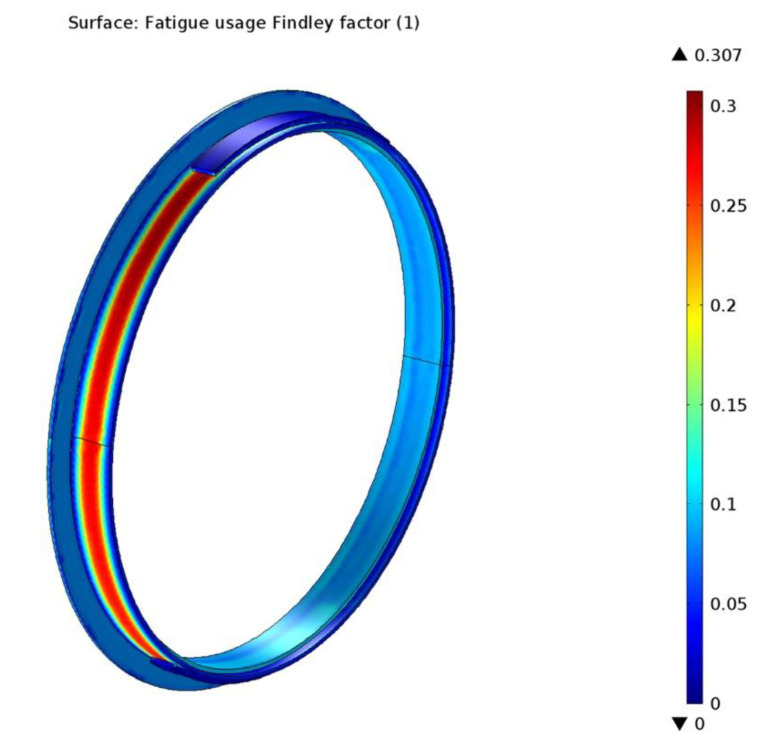
The Findley fatigue usage factor determined by simulation.

The results of the Findley simulation are presented in Figure 18. It shows that the highest fatigue usage factor has a value of 0.307 situated on the drum lining surface towards the exit point from under the brake shoe. A subunitary value means that the loads are below the fatigue limit of the material.

For the same materials, the Matake parameters in relation to the two endurance limits can be written as follows:

(22) $$f_{M}=\frac{\sigma_{R=-1}}{2}\left(1+k_{M}\right)$$

(23) $$f_{M}=\frac{\sigma_{R=0}}{2}\left(1+2\cdot k_{M}\right)$$

In a similar way the two equations were equalized to determine the Matake normal stress sensitivity coefficient (*k_M_*) and limit factor (*f_M_*) by solving them in Mathcad. The values obtained are *k_M_* = 0.404 and *f_M_* = 152.63 MPa, which are defined as the properties of materials for the COMSOL Matake Fatigue simulation menu, as seen in Table 7.

**Figure 19:**
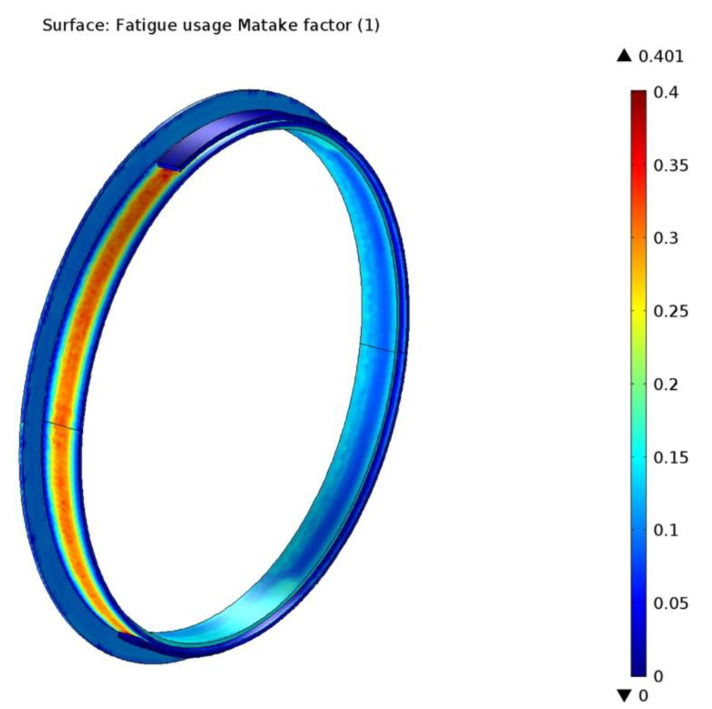
The Matake fatigue usage factor determined by simulation.

Next, in Figure 19 the result of the Matake simulation is presented in the form of the Matake usage factor, with a maximum value of 0.401, localized in the same area of the drum lining as in the case of the Findley simulation. This is normal, since the two criteria are very similar, the difference being that in the Matake criterion the plane with maximum shear stress range is taken as the critical plane. Again, a result smaller than 1 for the usage factor points to the conclusion that the fatigue limit of the material was not reached. The Matake method is considered less reliable than the Findley method in finding the critical plane, hence, the value of its usage factor is higher [46]. Therefore, the Findley method is usually recommended.

## 6. Conclusions

In this study, COMSOL Multiphysics is used for the determination of fatigue usage factors for Findley and Matake criteria in the case of a mine hoist drum brake system subjected to cyclic maneuver braking.

The approach was carried out step by step, as follows: (1) The geometric model of the brake was constructed using CAD tools; (2) the temperatures during braking were simulated using the heat transfer module; (3) this was followed by the determination of the thermally induced deformations and von Mises stresses using the solid mechanics module; (4) based on the results obtained and considering the endurance limits calculated for the material parameters of the brake, the usage factors resulted from the Findley and Matake fatigue studies.

Generally, stress-based fatigue models predict a fatigue usage factor, which is the fraction between the applied stress and the stress limit. In our case, for the two stress-based criteria (Findley and Matake) used to simulate the fatigue life of the components of a mine hoist brake system, the results showed a fatigue usage factor of 0.307 for the Findley criterion and a value of 0.401 for the Matake criterion. Since both values are under the critical value 1, this indicates that the stress limit has not been exceeded, thus, failure is not expected. The brake drum lining will hold for more than the expected fatigue life, which is either the service/replacement moment or the mandatory periodic technical inspection interval.

It was also determined that, during maneuver braking, the maximum temperature reached at the surface of the drum lining is lower than in the case of emergency braking, and also that the hottest point on the lining surface in the case of maneuver braking is different.

There are several possible future directions of work. The first would be to determine, in a similar way, the fatigue usage factors for the upgraded version of the MK5×2 mine hoist brake system which has a disc and pads layout, and to carry out a comparative analysis of the two braking solutions in terms of fatigue life evaluation. Another approach could be international research to investigate the brakes of other types of mine hoists used in Poland and Hungary. Finally, based on a similar modeling and simulation approach, we can investigate the fatigue life for the metallic structural elements of the mine hoist headframes, which are also subjected to cyclic stresses during the winding operation.

This type of step by step approach can be employed to study other industrial or vehicle brake types. The FEA software in general, and COMSOL Multiphysics in particular, allow for the testing of brakes and other components by numeric methods in terms of heat transfer, structural behavior, and allow us to make sure that the design/model stands up to external physical forces that appear during operation.

## Figures and Tables

**Table 1:** Features of the MK5×2 mine hoist brake system.

Feature	Value
Maximum braking moment	*M_f_* = 960 kN·m
Braking force	*F_f_* = 56 kN
Coefficient of friction of the drum-shoe couple	*μ* = 0.3
Drum brake external diameter	*D_f_* = 4800 m
Efficiency of the brake system	*η* = 0.95
Specific pressure of the shoes on the drum	*p* = 0.9 MPa
Rigidity of the springs	*Z* = 12.8 daN/mm
Maximum length of the springs	*L_max_* = 1295 mm
Minimum length of the springs	*L_min_* = 850 mm
Mass of the braking system	*m* = 5440 kg

**Table 2:** Hoisting parameters of the studied MK5×2 hoisting installation [3].

Parameter	Value
Hoisting depth	*H*_e_ = 913.4 m
Length of unloading curves	*h*_1_ = 3.5 m
Length of unloading chute (crawl space)	*h*_5_ = 2 m
Transport velocity	*v*_max_ = 12 m/s
Entrance velocity at the unloading chute	*v*_4_ = 0.5 m/s
Acceleration at start	*a*_1_ = 0.1 m/s^2^
Acceleration at exit of unloading curves	*a*_2_ = 0.7 m/s^2^
Deceleration at unloading chute	*a*_4_ = −1 m/s^2^

**Table 3:** Materials and properties for the studied mine hoist brake system components.

Property	Rope Wheel	Drum Lining	Brake Shoe	Unit
Density	7850	7000	2000	kg/m^3^
Poisson Ratio	0.33	0.25	0.25	-
Surface Emissivity	0.28	0.28	0.80	-
Specific Heat	475	420	935	J/(kg K)
Thermal Expansion Coefficient	12.3 × 10^−6^	11 × 10^−6^	12.3 × 10^−6^	K^−1^
Thermal Conductivity	44.5	50	8.7	W/(m K)
Yield Strength	6.2 × 10^8^	3.5 × 10^8^	2.93 × 10^8^	N/m^2^
Young Modulus	200 × 10^9^	140 × 10^9^	140 × 10^9^	Pa
Material	Alloy steel	Steel	Non asbestos composite	-

**Table 4:** Simulation parameters definition in COMSOL Multiphysics.

Name	Expression	Value	Description
mu	0.3	0.3	Coefficient of friction
v0	12 [m/s]	12 m/s	Initial speed
a0	−1 [m/s^2^]	−1 m/s^2^	First acceleration
a1	−0.1 [m/s^2^]	−0.1 m/s^2^	Second acceleration
t_brake_start	1 [s]	1 s	Braking time (start)
t_brake_end_1	t_brake_start + 11.5	12.5 s	Theoretical braking time (end)
t_brake_end_2	t_brake_end_1 + 8.36	20.86 s	Braking time (end)
t_STOP	t_brake_end_2 + 5	25.86 s	Simulation STOP
t_end	t_STOP + 20	45.86 s	Total simulation time
m_car	260,000 [kg]	2.6 × 10^5^ kg	Mass
T_air	300 [K]	300 K	Temperature, air
step	0.1 [s]	0.1 s	Step iteration
r_wheel	2.4 [m]	2.4 m	Drum radius

**Table 5:** Definition of the velocities for the COMSOL simulation as functions.

Start	End	Function
0.5	t_brake_start	v0
t_brake_start	t_brake_end_1	v0 + a0*(t − t_brake_start)
t_brake_end_1	t_brake_end_2	v0 + a0*(t_brake_end_1 − t_brake_start)
t_brake_end_2	t_STOP	v0 + a0*(t_brake_end_1 − t_brake_start) + a1*(t − t_brake_end_2)
t_STOP	t_end	0.0001

**Table 6:** Definition of material properties for the Findley fatigue simulation.

Property	Variable	Expression	Unit	Size
Normal stress sensitivity coefficient	k_Findley	0.208	1	1 × 1
Limit factor	f_Findley	126.57 × 10^6^	Pa	1 × 1

**Table 7:** Definition of material properties for the Matake Fatigue simulation.

Property	Variable	Expression	Unit	Size
Normal stress sensitivity coefficient	k_Matake	0.404	1	1 × 1
Limit factor	f_Matake	152.63 × 10^6^	Pa	1 × 1

## Data Availability

Not applicable.
